# Localizing evoked and induced responses to faces using magnetoencephalography

**DOI:** 10.1111/ejn.12520

**Published:** 2014-03-12

**Authors:** Gavin Perry, Krish D Singh

**Affiliations:** Cardiff University Brain Research Imaging Centre (CUBRIC), School of Psychology, Cardiff University70 Park Place, Cardiff, CF10 3AT, Wales, UK

**Keywords:** face perception, gamma-band, M170, source localization

## Abstract

A rich pattern of responses in frequency, time and space are known to be generated in the visual cortex in response to faces. Recently, a number of studies have used magnetoencephalography (MEG) to try to record these responses non-invasively – in many cases using source analysis techniques based on the beamforming method. Here we sought both to characterize best practice for measuring face-specific responses using MEG beamforming, and to determine whether the results produced by the beamformer match evidence from other modalities. We measured activity to visual presentation of face stimuli and phase-scrambled control stimuli, and performed source analyses of both induced and evoked responses using Synthetic Aperture Magnetometry. We localized the gamma-band response to bilateral lateral occipital cortex, and both the gamma-band response and the M170-evoked response to the right fusiform gyrus. Differences in the gamma-band response between faces and scrambled stimuli were confined to the frequency range 50–90 Hz; gamma-band activity at higher frequencies did not differ between the two stimulus categories. We additionally identified a component of the M220-evoked response – localized to the parieto-occipital sulcus – which was enhanced for scrambled vs. unscrambled faces. These findings help to establish that MEG beamforming can localize face-specific responses in time, frequency and space with good accuracy (when validated against established findings from functional magnetic resonance imaging and intracranial recordings), as well as contributing to the establishment of best methodological practice for the use of the beamformer method to measure face-specific responses.

## Introduction

The cortical basis of face perception has been a topic of interest for a number of decades. Early work based on primate neurophysiology provided evidence that there might be identifiable cortical regions devoted specifically to the processes of face perception (Gross *et al*., 1969, 1972). The introduction of functional magnetic resonance imaging (fMRI) allowed the identification of an extended network of face processing regions, including the fusiform gyrus, lateral occipital cortex and the superior temporal sulcus (for a review see Haxby *et al*., 2000). The relatively poor temporal resolution of the fMRI blood oxygen-level dependent (BOLD) signal makes the technique ill-suited to measuring the temporal dynamics of cortical processing, however. This is unfortunate as intracranial electroencephalography (EEG) in humans has revealed a rich pattern of both evoked and induced responses to faces (Allison *et al*., 1999; McCarthy *et al*., 1999; Lachaux *et al*., 2005; Barbeau *et al*., 2008; Tsuchiya *et al*., 2008; Fisch *et al*., 2009; Engell & McCarthy, 2010, 2011; Vidal *et al*., 2010; Privman *et al*., 2011; Davidesco *et al*., 2013).

Magnetoencephalograpy (MEG) currently offers the best potential for the non-invasive study of face processing with high temporal resolution. Of particular interest are the gamma-band response – which has been shown to involved in the processing of facial emotion (Luo *et al*., 2007, 2009; Maratos *et al*., 2012) and appears to be reduced in prosopagnosia (Dobel *et al*., 2011) and in other conditions which are characterized by face processing deficits such as autism (Wright *et al*., 2012) and schizophrenia (Grützner *et al*., 2013) – and the M170 event-related field (ERF) – which is known to be face-specific (Sams *et al*., 1997; Halgren *et al*., 2000; Liu *et al*., 2000) and may also be altered in prosopagnosia (Harris *et al*., 2005; Dobel *et al*., 2008; Prieto *et al*., 2011; Rivolta *et al*., 2012).

Many of these previous studies have made use of beamforming, a well-established technique for MEG source localization that has been used extensively to localize visually evoked and induced responses (see for instance Adjamian *et al*., 2004; Hall *et al*., 2005a,b; Hoogenboom *et al*., 2006; Muthukumaraswamy *et al*., 2010). However, the choice of various aspects of the beamformer analysis – in particular the choice of time and frequency windows of interest and the choice of relevant statistical comparisons – can have a critical impact on the results found using the technique. Yet we are not aware of any general attempt to characterize best practice for measuring face-specific responses using MEG beamforming, nor of any attempt to determine whether the results produced by the beamformer match evidence from other modalities such as intracranial recordings or fMRI.

Here we show that the MEG beamformer is able to localize responses to faces in time, frequency and space in a manner that is consistent with findings from other modalities. In doing so we demonstrate that, particularly for the gamma-band response, statistical comparison against a control stimulus (phase-scrambled faces) and precise choice of frequency bandwidth are critical to accurately finding face-specific responses.

## Materials and methods

### Participants

The participants were 20 volunteers (age range: 21–43 years, mean: 29 years) with normal or corrected-to-normal vision (based on self-report). The participant sample contained equal numbers of males and females. Each participant gave written consent to take part in the study in accordance with The Code of Ethics of the World Medical Association (Declaration of Helsinki). All procedures were approved by the ethics committee of the School of Psychology, Cardiff University.

### Stimuli

Face stimuli were 30 images each showing a front profile view of one of 15 females and 15 males, taken from the ECVP Utrecht face set (available at http://pics.psych.stir.ac.uk/zips/utrecht.zip). Images were 576 × 768 pixels in size and converted to 8-bit greyscale prior to use.

Control stimuli were created by phase scrambling each of the 30 images. Phase scrambling was achieved by performing a two-dimensional (2D) Fourier transform of each of the images, removing the phase information and replacing it with the corresponding phases taken from a white noise image of equal size to the original stimulus, and then performing the inverse Fourier transform to create the scrambled image. As phase scrambling can produce pixel intensities outside of the displayable range, pixel intensities of the scrambled images were linearly normalized (by subtraction of the minimum intensity and division by the sum of the minimum and maximum intensities) to within the displayable range. An identical normalization (that is using the minimum and maximum values taken from the matching scrambled image) was then applied to each of the original face stimuli to ensure that the low-level stimulus properties (i.e. the 2D Fourier amplitude spectrum, and the mean and standard deviation of the pixel intensities) were matched between each face stimulus and its scrambled counterpart. A new set of scrambled stimuli were generated for each participant.

### Data acquisition

Whole-head MEG recordings were made in 2.5-s epochs centred around the onset of each stimulus using a 275-channel CTF radial gradiometer system at a sampling rate of 1200 Hz. An additional 29 reference channels were recorded for noise cancellation purposes, and the primary sensors were analysed as synthetic third-order gradiometers (Vrba & Robinson, 2001). Two of the 275 channels were turned off due to excessive sensor noise, and a third sensor was turned off for the same reason during an annual system service which occurred part way through the study.

All stimuli were presented centrally on a mean grey background using a gamma-corrected Mitsubishu Diamond Pro 2070 CRT monitor with a screen resolution of 1024 × 768 pixels and a refresh rate of 100 Hz. The monitor was viewed from a distance of 2.1 m, with stimulus images subtending 8.3° × 6.1° of visual angle. In each trial participants viewed a white fixation cross for 1 s followed by one of the 60 stimulus images for 1 s, then a further period of fixation of random duration selected at each trial from a uniform distribution between 600 and 900 ms. Each stimulus was presented eight times during the experiment, leading to a total of 480 trials. Participants performed a change detection task in which, at the start of a pseudorandomly selected 10% of trials, the fixation cross turned red. The cross remained red until participants responded with a right index finger button press, with the trial ‘proper’ then beginning after a 500-ms delay. The experimental paradigm was implemented in Matlab (The Mathworks, Natick, MA, USA) using the Psychophysics Toolbox (Brainard, 1997; Pelli, 1997; Kleiner *et al*., 2007).

At the start and end of each session each participant's head position was localized by means of three fiducial coils attached to specific anatomical locations on the scalp (nasion and left and right pre-auricular). The maximal head displacement between the start and end of a session in any participant was 1.2 cm. Fiduciary locations were verified afterwards using high-resolution digital photographs. These locations were then located on previously acquired anatomical MRIs of each participant for the purposes of source analysis.

### Data analysis

Data were manually inspected offline, and trials containing artefacts related to excessive muscle or head movements were excluded from analysis.

For evoked (but not induced) analysis data were bandpass filtered at 1–30 Hz (all filtering in the study was performed using third-order bi-directional IIR Butterworth filters, with the exception of filtering for source analysis where fourth-order filters were used). Evoked time windows of interest (TWOIs) were identified by averaging data across all trials for each participant to create an ERF timeseries for each sensor, then averaging these timeseries across participants to create a grand average ERF timeseries. These grand average timeseries were then baseline corrected against the 150 ms prior to stimulus onset, and the global field power (GFP) was calculated at each timepoint by calculating the root mean square (RMS) of the amplitude across sensors. TWOIs were then defined based on the local minima of the GFP timeseries.

For induced analysis, time–frequency spectrograms were calculated for each sensor (excluding the sensor which was turned off part way through the study – see Data acquisition section above) by bandpassing the data using filters of 4 Hz bandwidth centred on 2–160 Hz in 1 Hz steps and then using the Hilbert transform to compute the analytical signal at each frequency. Time–frequency data were than averaged across all trials for each participant. The magnitude of the response at each time–frequency point was calculated as the percentage amplitude change relative to the mean amplitude at that frequency during the time period −1 to 0 s relative to stimulus onset. To determine time–frequency windows of interest (TFWOIs), we used Wilcoxon signed-rank tests to identify time × frequency × sensor points for which the median magnitude across participants differed from zero. To correct for multiple comparisons we adjusted the alpha level of these tests to produce a false discovery rate of 0.05 (Genovese *et al*., 2002). TFWOIs were then determined based on contiguous time–frequency regions that were significant over a substantial number of sensors (see Results).

Source analysis was performed using the Synthetic Aperture Magnetometry (SAM) beamformer algorithm, which is described in detail elsewhere (Robinson & Vrba, 1999; Vrba & Robinson, 2001; Hillebrand *et al*., 2005). SAM operates by constructing an adaptive spatial filter based on a combination of a forward model and the data covariance matrix. Each participant had a previously acquired structural MRI, and a multiple local-spheres forward model (Huang *et al*., 1999) was derived by fitting spheres to the brain surface extracted by the Oxford fmrib Software Library's Brain Extraction Tool (Smith, 2002). Data covariance matrices were calculated from the full dataset for each participant after bandpass filtering at either 1–30 Hz (in the case of the evoked analysis) or at specific frequencies of interest (for the induced analyses). For evoked analyses we followed the method suggested by Robinson (2004) and Cheyne *et al*. (2006) and calculated data covariance from unaveraged data. Volumetric images of source power were then derived at 4 mm isotropic resolution by projecting each participant's raw data through the corresponding spatial filters.

For each analysis, individual *t*-statistical images were calculated at each voxel for each participant using Welch's *t*-test for unequal variances: 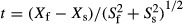, where *X*_f_ and *X*_s_ are the response measures to faces and scrambled stimuli, respectively, and *S*_f_ and *S*_s_ are the standard errors of those measures. For evoked analyses *X* was the mean power of the evoked time series within the time window of interest and *S* was estimated using a jackknife procedure (due to power being calculated after averaging over trials). For induced analyses *X* was the mean of the bandpass-filtered analytical time signal (generated using the Hilbert transform) averaged across trials within the time window of interest, and *S* was estimated from the standard deviation of the measure. All response measures, *X*, were baseline corrected (against the 150 ms prior to stimulus onset for evoked responses, and for time windows ending 200 ms prior to stimulus onset and of equal length to the time window of interest for induced responses).

To perform group-level analyses, individual-level *t*-statistic images were then normalized using FLIRT (www.fmrib.ox.ac.uk/analysis/research/flirt) into MNI template space using an affine transform. A mask was used to exclude voxels outside of the brain.

The locations of any group-level differences in the response to faces and scrambled stimuli were then found using permutation testing (Nichols & Holmes, 2001; Singh *et al*., 2003). For each analysis, group-level *t*-statistics were calculated for each voxel using participant-level *t*-statistics as the measure. Due to the focal nature of sources found in the evoked analysis, variance smoothing was applied using a Gaussian kernel (σ = 12 mm) as this substantially improved the statistical power of the analysis. The distribution of group-level *t*-statistics was then estimated at each voxel by randomly flipping the signs of the individual-level test statistic (equivalent to randomly swapping the condition labels between face and scrambled stimuli) and recalculating the group-level *t*-statistic. Five thousand such permutations were generated and used to determine voxelwise *P*-values for the group-level difference between the response to faces and scrambled stimuli. Corrections for multiple comparisons were then performed by thresholding using the maximum test statistic.

For virtual sensor analyses, locations of interest were identified for each analysis by finding peaks in individual participants' *t*-statistical images. Sensor-level data were then spatially filtered using the beamformer weights at the location of interest to create a single timeseries per trial per participant per analysis. Evoked and induced analyses were then carried out in the manner outlined above for sensor-level data, but separately for face and scrambled trials. As SAM is a scalar beamformer – with source orientation set by a non-linear search – the polarity of evoked timeseries is arbitrary (due to source direction and the sign of source moment being interchangeable) and so the polarities of all evoked timeseries in virtual sensor analyses were set such that the maximal deflection was positive within the TWOI.

## Results

### Induced responses

To perform source-level analysis of the induced responses, we first found TFWOIs in the sensor-level data. Rather than calculate TFWOIs based on time-frequencies at which the responses to face and scrambled stimuli most differed – which would potentially create a bias in favour of finding differences in the same time–frequency windows at the source level (and could constitute an instance of the statistically invalid practise of ‘double dipping’) – we instead calculated the time–frequency response at each sensor for each participant across all trials. At each time × frequency × sensor point we tested against a median of zero across participants using a Wilcoxon signed-rank test (corrected for multiple comparisons using a false discovery rate of 0.05), to determine at which time–frequency points the response to the stimuli differed from zero independently of condition.

**Figure 1:**
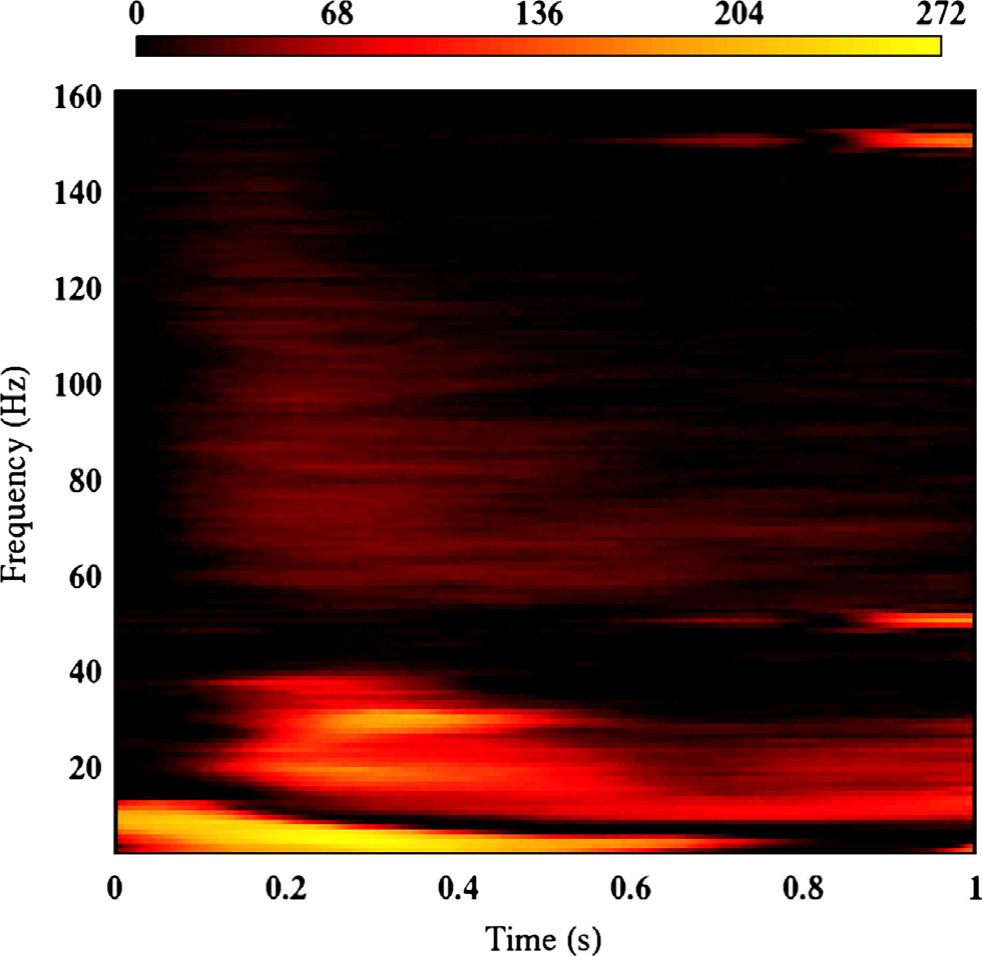
Plot of the number of sensors for which the response to stimuli (averaged across all trials of both conditions) significantly differed from zero for each time × frequency point. The colour scale depicts number of significant sensors.

Figure1 shows the number of significant sensors at each time × frequency point. Based on this figure we determined three TFWOIs: 0–6 Hz, 100–600 ms; 15–40 Hz, 150–500 ms; 55–120 Hz, 100–400 ms. We refer to these time windows as: delta/theta, beta and gamma, respectively.

**Figure 2:**
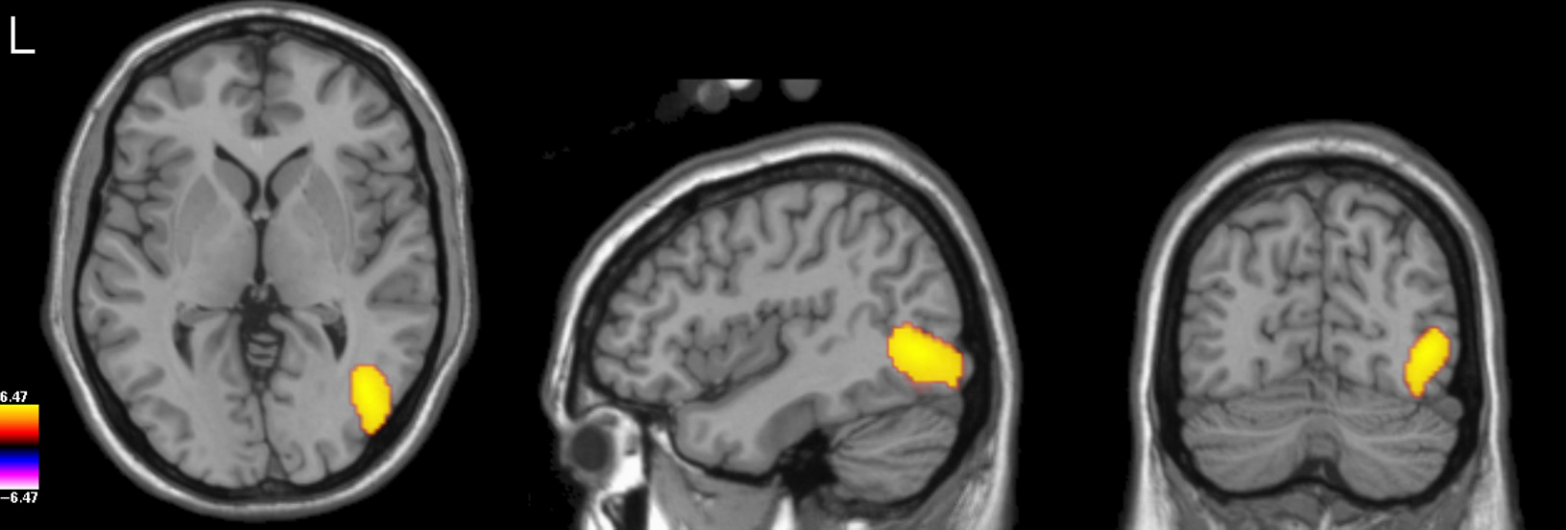
Group-level *t*-statistical image (thresholded at *P* < 0.05 FWE) of the difference in response to faces vs. scrambled stimuli in the gamma TFWOI (55–120 Hz, 100–400 ms), overlaid on the template brain.

We then used the SAM beamformer to produce volumetric images of source power for each condition (face and phase scrambled). Group-level analyses of within-participant differences between the two conditions revealed no significant difference in the delta/theta or beta TFWOIs. In contrast, significant differences were found for the gamma TFWOI (Fig.2) with enhanced response amplitudes found for faces relative to scrambled stimuli in a region of right lateral occipital cortex. The most significant voxel occurred on the middle occipital gyrus (Talaraich coordinates: + 43.2, −71.3, + 1.0), close to the proposed location of the occipital face area (OFA; see table 1 of Pitcher *et al*., 2011, for a summary of Talairach coordinates of right OFA from 14 fMRI studies – the coordinates of OFA reported here are within 12 mm of the mean location across these studies).

**Figure 3:**
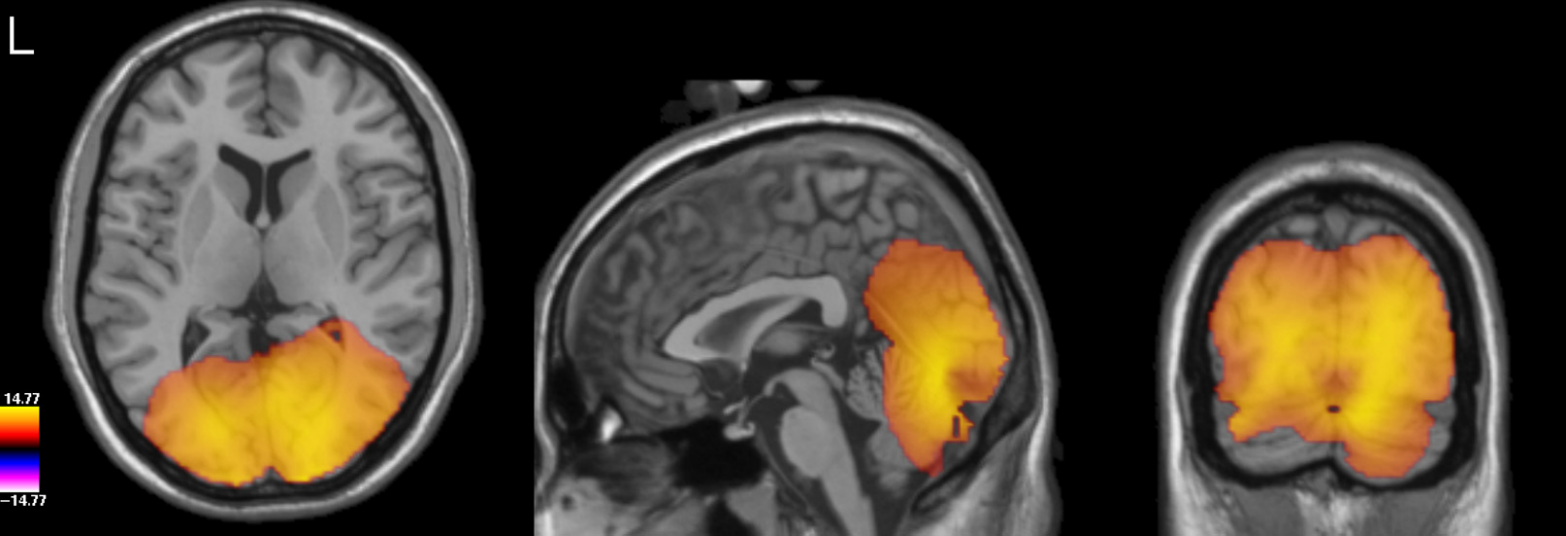
Group-level *t*-statistical image (thresholded at *P* < 0.001 FWE) of the difference in response to faces vs. baseline (−500 to −200 ms) in the gamma TFWOI (55–120 Hz, 100–400 ms), overlaid on the template brain.

In many previous studies in which MEG beamforming has been used to localize the gamma-band response to faces, control stimuli were not used and instead contrasts were performed between the response to faces vs. the pre-stimulus baseline. To compare our findings with this prior work, we repeated our gamma-band analysis using the same parameters as above, but contrasting the response to face stimuli with the response present in the stimulus baseline period rather than with the response to scrambled stimuli. Highly significant increases in gamma power to faces relative to baseline were found widely throughout occipital cortex (Fig.3), with the most significant voxels being found bilaterally around the occipital pole (Talairach coordinates −L: −11.0, −105.4, −3.0; R: 19.1, −103.4, 5.0). In contrast, no peak was present in the statistical parametric map around the middle occipital gyrus (or any other nearby location) as was found in the face vs. scrambled stimulus comparison. Instead, given their localization of the strongest effect to striate and/or neighbouring areas of extrastriate cortex, the findings from the face vs. baseline comparison suggest strongly that the largest gamma differences are generated by differences in image contrast between stimulus and baseline and not due to the presence (or absence) of a face. This has implications for previous studies of the gamma-band response to faces based on beamforming (see Discussion).

We were interested to explore the time course and frequency characteristics of the response found in the face vs. scrambled stimulus contrast, and so performed a virtual sensor analysis. For each participant we inspected the *t*-statistical image of the difference between conditions, and found the largest local maxima in the right lateral occipito-temporal region (two participants were excluded from this analysis due to absence of a local maximum in the area of interest). We then used the beamfomer weights to generate virtual sensor timeseries at this location.

**Figure 4:**
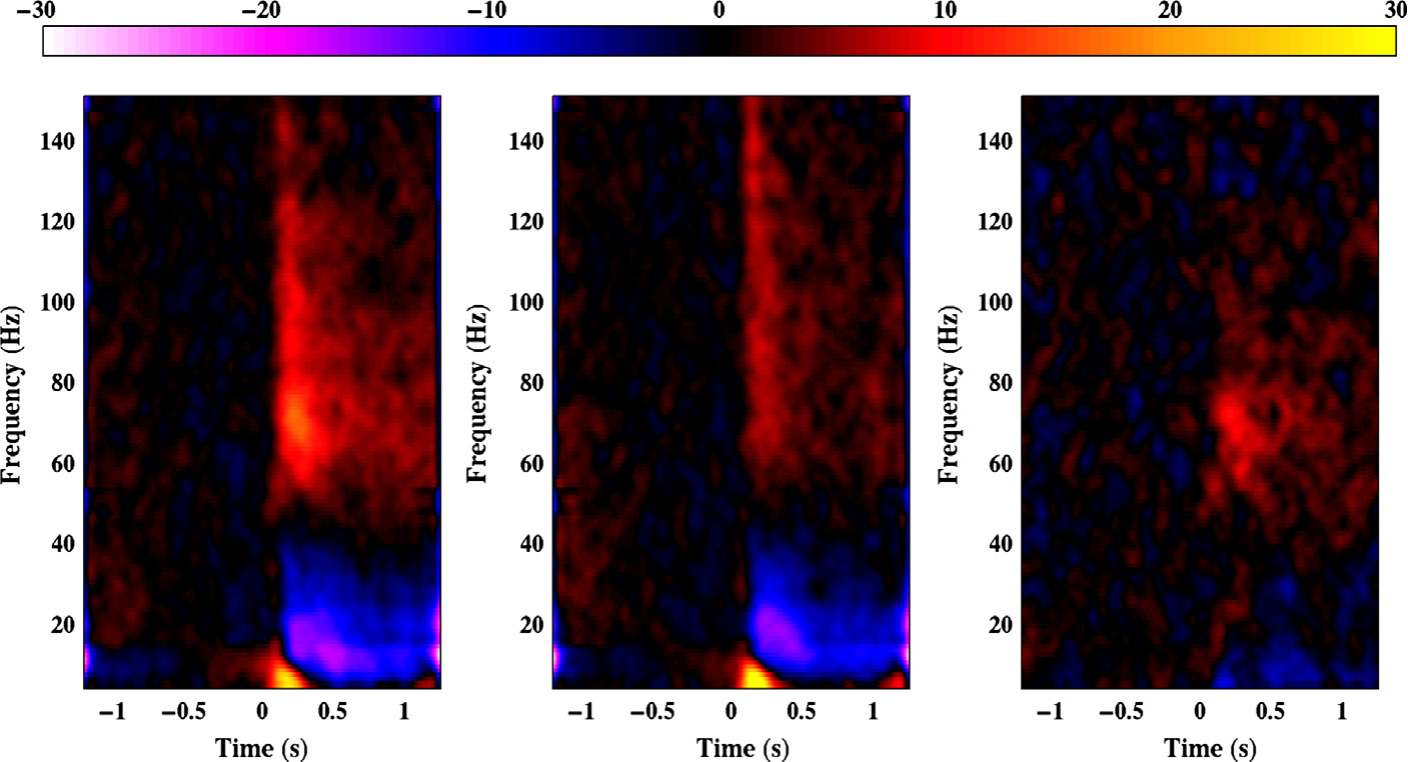
Group average spectrograms depicting the virtual sensor responses to face (left panel) and scrambled stimuli (middle panel), and the difference between the two responses (right panel). The colour scale depicts amplitude in percentage relative to baseline (−1 to 0 s).

**Figure 5:**
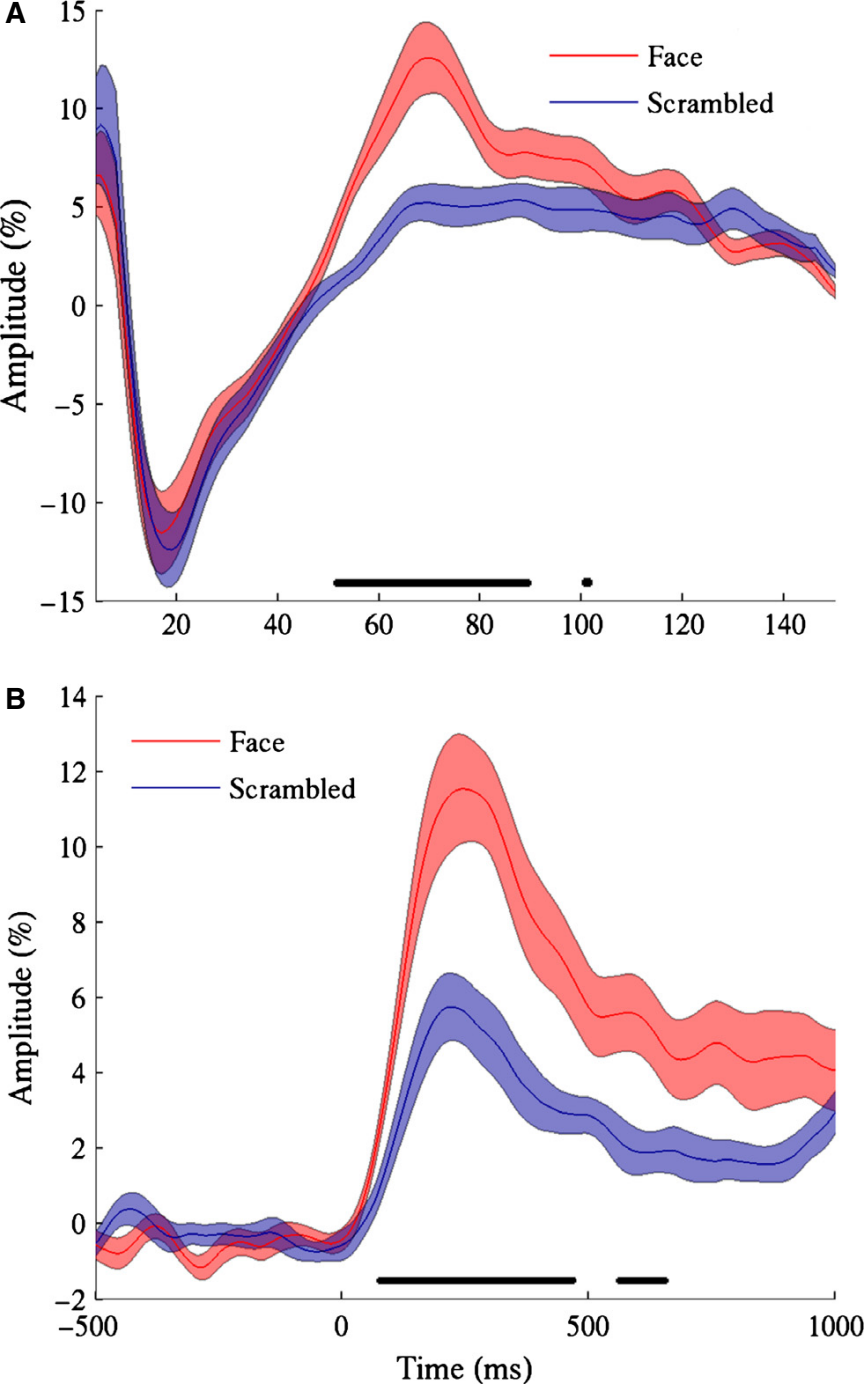
(A) Group mean (± SE) amplitude spectra of the response from 100 to 400 ms following stimulus onset for both faces and scrambled stimuli. Spectra were smoothed with a uniform kernel of width 9 Hz to reduce spectral noise. (B) Group mean (± SE) amplitude of the gamma (55–120 Hz) response against time for both faces and scrambled stimuli. In both plots the dark bars above the *x*-axis depict frequencies/times at which the responses to face and scrambled stimuli were significantly different (Wilcoxon signed-rank test, *P* < 0.01 FDR).

Figure4 demonstrates that, while both the face and the scrambled stimuli induce a broadband gamma response extending from around 50 Hz up to at least 150 Hz, there is little difference between conditions for frequencies above 80 Hz. This is confirmed in Fig.5A, which shows the group average amplitude spectrum across the time period 100–400 ms for both conditions. Significant differences between the two spectra were confined to the range of approximately 50–90 Hz – a narrower frequency bandwidth than used in our initial gamma TFWOI.

The time course of the gamma response was similar for both conditions (Fig.5B), with a rapid rise in amplitude soon after stimulus onset, reaching a peak at around 220 ms for scrambled stimuli and 245 ms for face stimuli, followed by a decrease to a sustained response with a lower amplitude. The increased amplitude to faces vs. scrambled stimuli was present within the first 100 ms, but was largest around the response peak, although a moderate amplitude enhancement to faces remained throughout the stimulus presentation.

**Figure 6:**
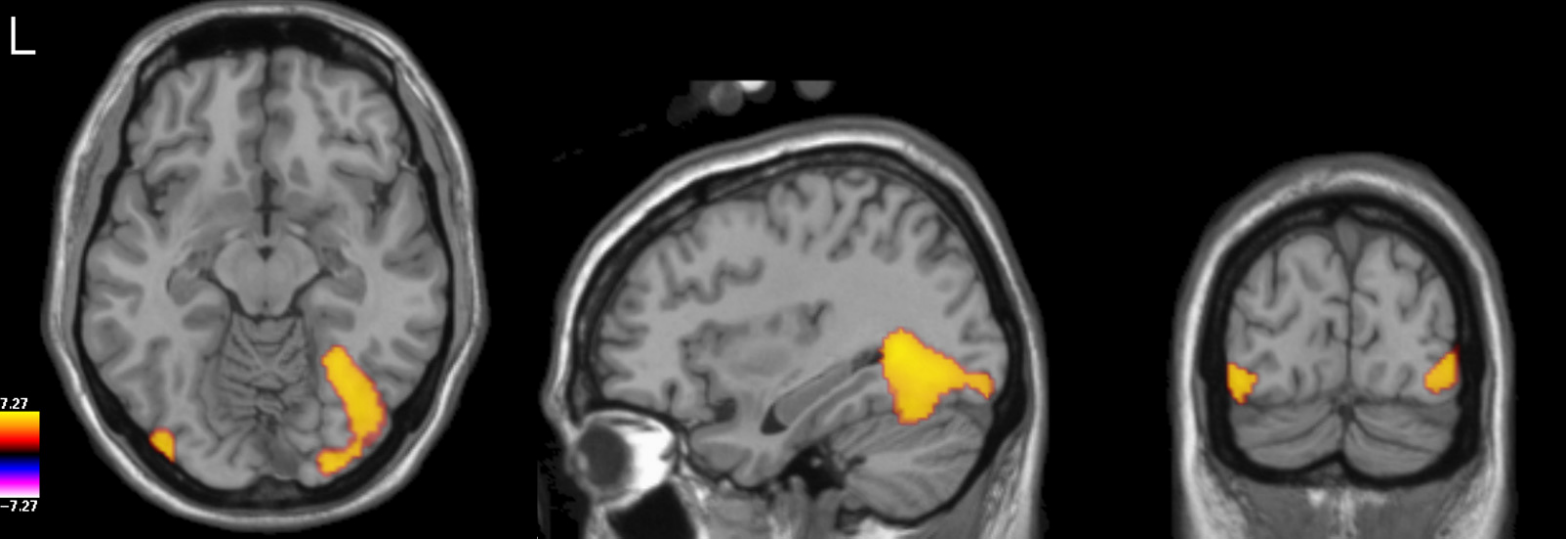
Group-level *t*-statistical image (thresholded at *P* < 0.05 FWE) of the difference in response to faces vs. scrambled stimuli in the refined gamma TFWOI (50–90 Hz, 100–450 ms), overlaid on the template brain.

These findings suggest that our gamma TFWOI might not have been optimal to find differences between conditions. Thus, we reran our group-level volumetric analysis using a new gamma TFWOI: 50–90 Hz, 100–450 ms (see Fig.6). As before, significant differences were present in right lateral occipital cortex, but this time also extended more anteriorly across the ventral surface of the cortex, extending to the fusiform gyrus (Talaraich coordinates: 35.1, −65.3, −13) around the posterior part of the fusiform face area (FFA). Significant differences were also found in left lateral occipital cortex (Talaraich coordinates: −41.2, −87.3, −7.0). Thus, by optimizing our TFWOI we were able to increase the statistical sensitivity of our analysis and uncover regions of significant difference between conditions that were not present in our initial analysis.

**Figure 7:**
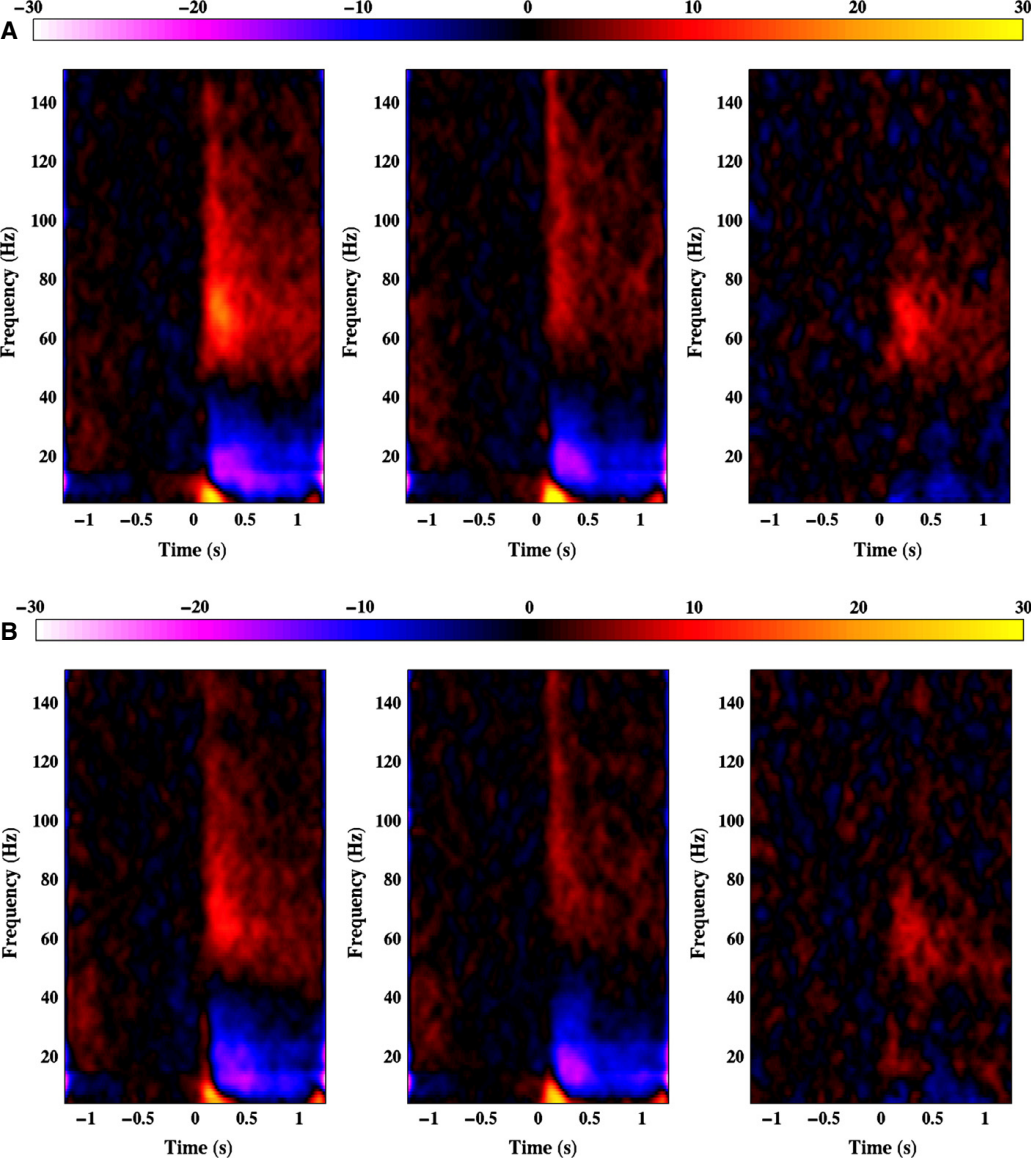
(A) Group average spectrograms depicting the right hemisphere virtual sensor responses to face (left panel) and scrambled stimuli (middle panel), and the difference between the two responses (right panel), using weights from the 50–90 Hz gamma analysis. (B) As for A but for the left hemisphere virtual sensors.

To determine whether our use of a different frequency bandwidth to calculate the beamfomer weights affected the virtual sensor analysis, we re-ran the analysis using the new weights – this time using local maxima from both the right and the left lateral occipito-temporal cortices (one participant was excluded due to an absence of local maxima in the corresponding areas, while four further participants contributed only one virtual sensor: two for the right hemisphere, and two for the left). The results shown in Fig.7 demonstrate that the time–frequency characteristics of the response to both faces and scrambled stimuli (and the difference between them) were not substantially different from those found in the initial virtual sensor analysis (shown in Fig.4). The figure also demonstrates that the time–frequency characteristics of the left hemisphere response were highly similar to those found in the right hemisphere, but with a weaker response amplitude in the left hemisphere.

### Evoked responses

**Figure 8:**
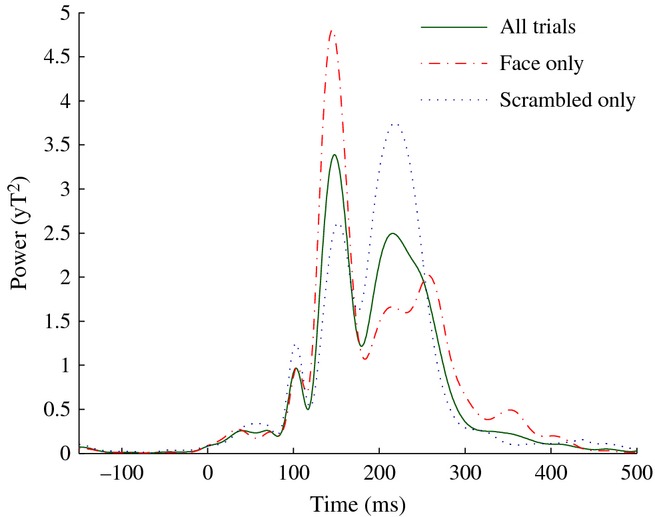
Global field power of the grand average ERF across all trials, and for face and scrambled trials separately.

To define TWOIs for the analysis of evoked responses, we calculated the omnibus (i.e. averaged across all trials) GFP of the group average sensor-level ERFs. Based on local minima in the omnibus GFP (Fig.8), we defined three TWOIs: 83–116 ms (M100), 116–181 ms (M170) and 181–300 ms (M220).

As with the induced responses, we then performed group-level volumetric analyses of differences in source power between the face and scrambled conditions. Significant differences between conditions were found in the M170 and M220 time windows, but not in the M100 window.

**Figure 9:**
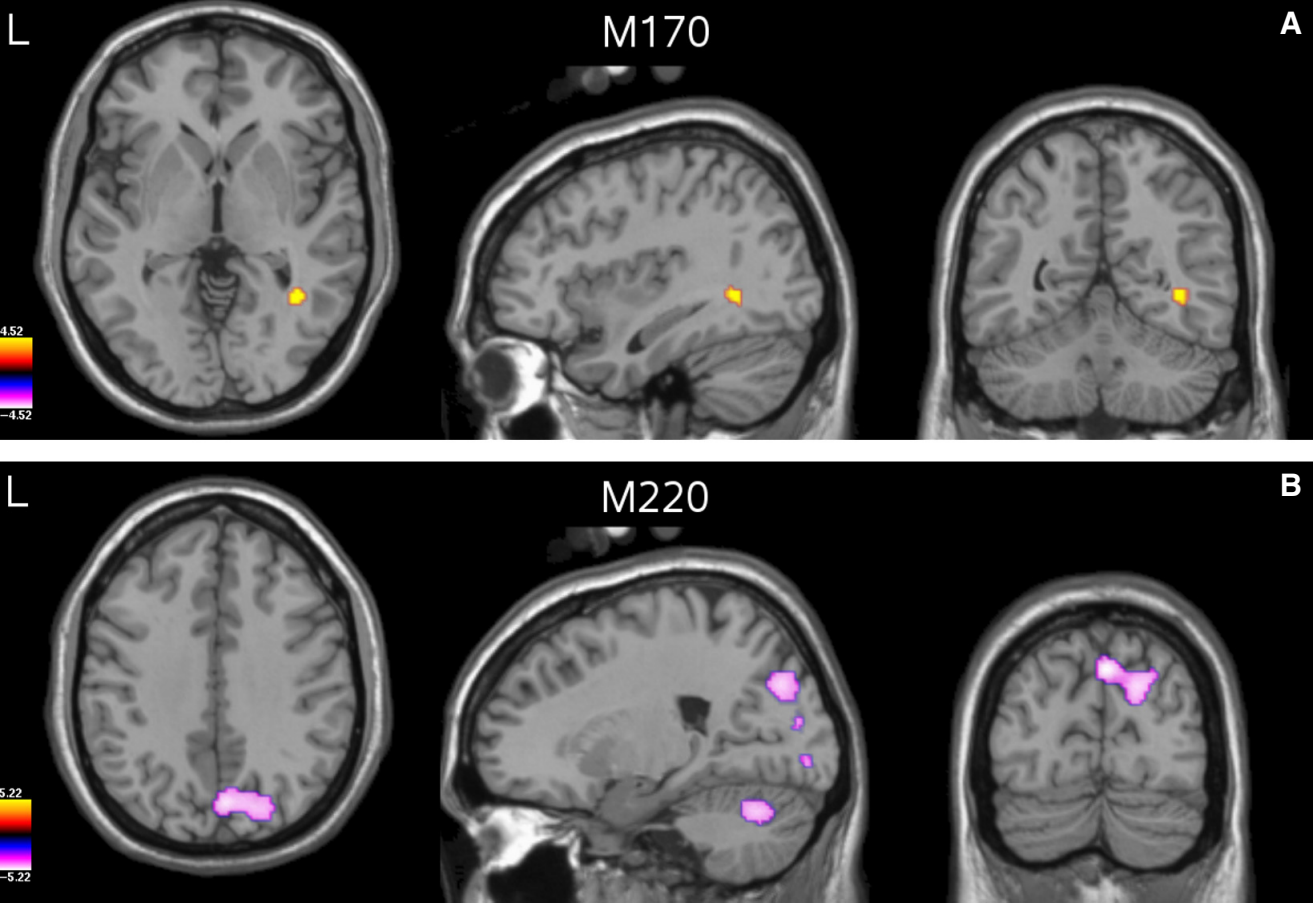
(A) Group-level *t*-statistical image (thresholded at *P* < 0.05 FWE) of the difference in response to faces vs. scrambled stimuli in the M170 analysis, overlaid on the template brain. (B) As for A but for the M220 analysis.

**Figure 10:**
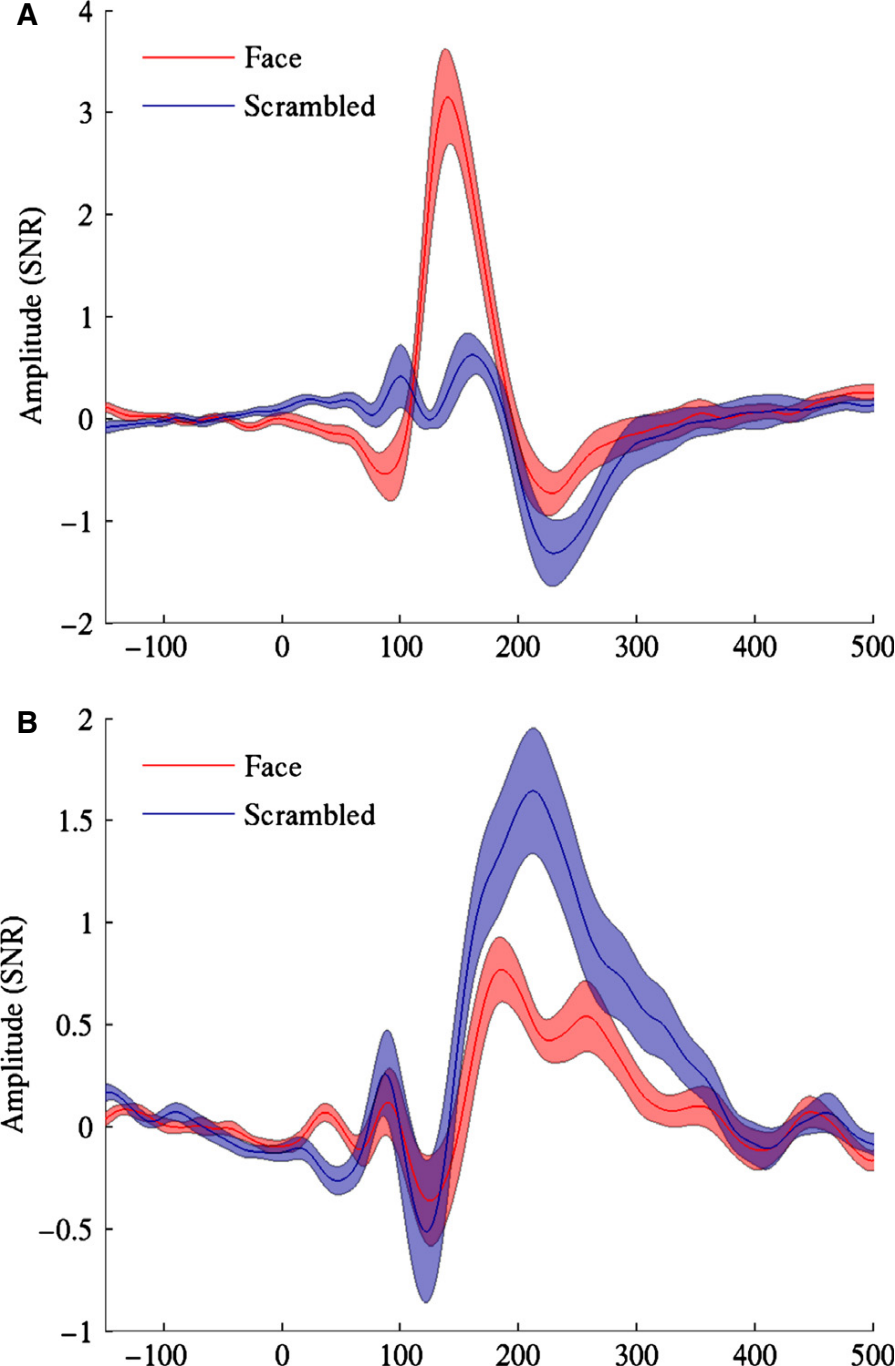
(A) Group mean (± SE) ERF from the M170 virtual sensor analysis. (B) As for A, but for the M220 analysis.

For the M170 analysis (see Fig.9A) a single area was found in which the faces produced enhanced source power relative to scrambled stimuli. This was situated in a posterior region of ventro-temporal cortex, close to the region of fusiform gyrus for which we found a gamma amplitude enhancement for faces, but shifted somewhat anteriorally and superiorally so that the most significant voxel occurred in the adjacent white matter (Talairach coordinates: + 37.1, −55.2, −1.0). To explore the time course of this effect we performed a virtual sensor analysis of this response by finding local maxima in the approximate location of this effect in individual-level images of between-condition differences (two participants were excluded from this analysis due to absence of a local maximum in the area of interest). Consistent with our attribution of this effect to the M170 ERF, the major difference between conditions was a prominent peak around 140 ms to face stimuli, which was almost entirely absent when the stimuli were scrambled (Fig.10A).

For the M170 analysis (see Fig.9A) a single area was found in which the faces produced enhanced source power relative to scrambled stimuli. This was situated in a posterior region of ventro-temporal cortex, close to the region of fusiform gyrus for which we found a gamma amplitude enhancement for faces, but shifted somewhat anteriorally and superiorally so that the most significant voxel occurred in the adjacent white matter (Talairach coordinates: + 37.1, −55.2, −1.0). To explore the time course of this effect we performed a virtual sensor analysis of this response by finding local maxima in the approximate location of this effect in individual-level images of between-condition differences (two participants were excluded from this analysis due to absence of a local maximum in the area of interest). Consistent with our attribution of this effect to the M170 ERF, the major difference between conditions was a prominent peak around 140 ms to face stimuli, which was almost entirely absent when the stimuli were scrambled (Fig.10A).

For the M220 analysis (Fig.9B) regions were found in which source power to faces was decreased relative to that produced by scrambled stimuli. The most significant difference was found in a region of the parieto-occipital sulcus (Talairach coordinates: + 3.0, −77.3, + 41.0) extending rightwards into the cuneus, with an additional smaller region occurring on the medial surface of the lingual gyrus. A significant region was also found in the dorsal part of the right cerebellum, but as we consider it unlikely that the cerebellum would show differential activation to the two conditions in our study we consider that this was either a false positive finding or the mislocalization of activity stemming from the nearby ventral surface of the cortex. We again performed a virtual sensor analysis, this time based on the locations of local minima around the region of the parieto-occipital sulcus in individual-level images of differential source amplitude (one participant was excluded due to the absence of a local minimum in this region). Figure10B illustrates the presence of a substantial peak around 210 ms (consistent with its identification as part of the M220 component), which was present for scrambled stimuli but largely attenuated for faces.

## Discussion

In this study we have explored the capabilities of MEG in conjunction with the SAM beamformer to localize face-specific cortical activity in frequency, time and space. The purpose of the study was both to demonstrate best practice for the use of the beamforming method to measure face-specific responses and to test the accuracy of these results against face-specific responses measured in other modalities. We will now discuss our results with respect to these aims, as well as the broader theoretical implications of our findings.

### Accuracy of source localization

One fundamental question mark over the use of MEG for localization is that source reconstruction is ill-posed: there is no unique solution to the MEG inverse problem, meaning that MEG source images cannot be guaranteed to accurately reflect the true state of cortical activity. In the case of face processing we have good prior knowledge of the expected sources of activity: a large body of fMRI studies have collectively mapped the cortical preference for faces and implicated two main regions: an area of the lateral occipital cortex, OFA, and an area of the fusiform gyrus, FFA (for review see Haxby *et al*., 2000). Moreover, a general tendency for face-specific activity to be right lateralized within these regions has also been described (e.g. Kanwisher *et al*., 1997; Pitcher *et al*., 2007). Therefore, the fact that in this study we have found gamma-band activity in broadly similar cortical areas and with a general degree of right lateralization at the group level (albeit that with some refinement to our time-window frequency of interest we also found activity in a region probably corresponding to left OFA) provides confidence that the beamformer reconstructed the spatial distribution of the gamma-band response with a reasonable degree of accuracy.

One further area found to be involved in face processing in fMRI studies, the superior temporal sulcus (STS), was not found to be active in this study. However, we note that the STS has been proposed to be involved in changeable aspects of faces, such as expression or gaze direction (Haxby *et al*., 2000), and thus might not have been expected to be strongly active in the current experiment in which static images with mostly neutral expressions were used. Consistent with this, previous MEG beamformer studies have localized gamma-band activity to STS when dynamic face stimuli have been used (Hagan *et al*., 2009; Lee *et al*., 2010).

One factor that we did not account for in our source localization was facial emotion in the stimuli used: the face database we used contained both neutral and happy faces and we did not distinguish between these emotions when selecting stimuli for use in the study. Thus, our data are from the presentation of a mixture of neutral and happy faces. However, we note that a meta-analysis of fMRI studies (Fusar-Poli *et al*., 2009) did not find substantial differences in localization of activity in the visual cortex to different facial emotions, and we therefore think it unlikely that our source localizations would have substantially differed if exclusively neutral or happy faces were used. We do note though that gamma-band differences in the response to different facial emotions have been reported (Luo *et al*., 2007, 2009; Maratos *et al*., 2012) albeit in activity generated in different areas of visual cortex than those reported here (see below: Comparison with previous studies – Gamma). Future work in which the effects of facial emotion are explicitly tested using the methodology outlined in this study will be necessary to test whether such effects were present in our data.

### Gamma-band response

As further evidence of the accuracy of our beamfomer reconstruction the time frequency characteristics of the gamma-band response found in our virtual sensor analyses (namely a broadband response in the gamma frequency range peaking around 240 ms; see Figs4 and 7) is highly consistent with that found in studies of intracranial electrophysiology in humans (Tsuchiya *et al*., 2008; Vidal *et al*., 2010; Privman *et al*., 2011; Davidesco *et al*., 2013). One interesting new finding in this study, however, is that not all of the face-induced gamma-band response is truly face-specific. Only the response within a narrower frequency range from around 50 to 90 Hz differed between faces and scrambled stimuli, with higher frequency components being common to both.

One potential caveat to this finding is that the power in MEG signals is known to scale in inverse proportion to (an exponent of) the frequency of the response. This could lead to data covariance calculations (and hence the beamformer source reconstruction) being dominated by signals at frequencies towards the lower end of the signal bandpass. Thus, our findings of differences only at the lower end of our gamma frequency bandwidth could be a result of this bias. To test this we re-ran our face vs. scrambled stimuli comparison at 100–150 Hz. We did not replicate any significant effects that were found in the 50–90 Hz bandwidth (or even find substantial, but non-significant, differences). Thus, we conclude that differences between stimuli were genuinely restricted to the 50–90 Hz frequency range.

Interestingly, studies in primates of the visual gamma response to grating stimuli have demonstrated a difference between high-frequency/broadband gamma, which appears to be generated by (or at least closely coupled to) local spiking activity, and low-frequency/narrowband gamma, which reflects a coherent oscillation within local field potentials across extended regions of cortex (Jia *et al*., 2011; Ray & Maunsell, 2011). Similarly, a recent MEG study has demonstrated the presence of separate high- and low-frequency components of the gamma-band that are differentially modulated by visual attention (Koelewijn *et al*., 2013).

The gamma response shown in Fig.5A appears to demonstrate both these components: in addition to the relatively narrowband response present for faces around 70 Hz, there is a broadband response in frequencies above 60 Hz which is of lower amplitude but common to both stimulus types. This would imply that the differences in response to faces and scrambled stimuli at our virtual sensor sites were not signalled by differences in mean firing rates, but were instead due to faces showing a greater propensity to induce a coherent gamma oscillation in local field potentials. This is consistent with theories that oscillations in the gamma frequency range play a critical role in the perception of coherent structure in an image through ‘feature binding’ and/or act as a mechanism to synchronize neuronal spiking to facilitate activation of downstream neurons (see Jensen *et al*., 2007 for more detailed discussion of these ideas). This finding should help to refine existing models of the face-specific gamma-band response (Privman *et al*., 2013).

### Beta- and delta/theta-band responses

In this study we have also explored the presence of face-specific responses in the beta frequency range (15–40 Hz) and in a lower frequency range (0–6 Hz), which we have dubbed delta/theta. We did not find any differences between faces and scrambled stimuli at these frequencies. Vidal *et al*. (2010) have demonstrated response selectivity between a group of visual stimulus categories, including faces, in the range 8–24 Hz at intracranial recording sites in bilateral occipito-temporal cortex, and thus we may have expected to find face-specific responses in our beta-band analysis. In contrast, Lachaux *et al*. (2005) failed to find beta-band modulations to upright vs. inverted faces, while Fisch *et al*. (2009) found that perception of backward masked faces was reflected in gamma- but not beta-band amplitude. Thus, the presence of differences in the beta frequency range appears to be sensitive to study design. As we are not aware of any intracranial studies that have used the current methodology (i.e. comparison of faces with matched control stimuli) we cannot independently validate our finding of a lack of effect in the beta-band (or in the range 0–6 Hz).

### Evoked responses

In addition to analysis of the induced response, we also analysed the evoked response to faces in the form of three previously described ERFs, the M100, M170 and M220. The M170, and its event-related potential (ERP) counterpart the N170, is a well-known evoked response which is enhanced to faces relative to other stimuli. Source localization methods have implicated all three of the face responsive areas mentioned above – FFA (Sams *et al*., 1997; Deffke *et al*., 2007; Henson *et al*., 2007), OFA (Halgren *et al*., 2000; Schweinberger *et al*., 2002) and STS (Itier & Taylor, 2004) – as potential sources of the M/N170. Here we localized the M170 to a region of ventral temporal cortex that was overlapping with gamma-band activity and which we attributed to FFA. The location of our peak difference for the M170 was within 13 mm of that reported in an intracranial study (Allison *et al*., 1999), suggesting that the beamfomer localization was reasonably accurate (although see below: Comparison with previous studies – M170).

The latency of the M170 peak – around 140 ms – was earlier than that reported in other MEG studies of face processing, which report latencies of around 160 ms (Sams *et al*., 1997; Halgren *et al*., 2000; Liu *et al*., 2000; Deffke *et al*., 2007), and substantially earlier than that found in intracranial recordings, which typically report latencies of around 200 ms (Allison *et al*., 1999, 2002; McCarthy *et al*., 1999; Engell & McCarthy, 2010, 2011; although see Barbeau *et al*., 2008 for latencies more consistent with the EEG/MEG data). However, the morphology of our response, dominated by a single large deflection present only for faces, was consistent with these prior studies. Thus, while the differences in latency between this study and others remain to be explained (and in the case of intracranial recordings may be due to medication effects in those undergoing a craniotomy), we are confident that the beamformer accurately reconstructed the time course of the evoked response.

We did not find any face-specific responses within the M100 time window. This stands in contrast to previous findings of an enhanced M100 response to faces relative to other stimulus categories (Liu *et al*., 2002). However, we note that in a study of the ERP counterpart – the P100 – which also used phase scrambled control stimuli, between-category response differences were found to be eliminated when differences in low-level image properties were taken into account (Rossion & Caharel, 2011). This again illustrates the importance of using matched control stimuli.

We did find one response that was enhanced to the scrambled stimuli relative to the faces: a parieto-occipital source of the M220 ERF. This finding was surprising: while the M220 is a response to faces that has been described in the MEG literature (Itier *et al*., 2006), the response was previously localized to ventral occipital rather than parieto-occipital sites. Moreover, we are not aware of any previous evidence that an evoked response with a preference for scrambled over unscrambled faces exists at this site, or any predictions that it should exist. This makes the finding difficult to interpret and means that future studies will be necessary to clarify this effect. However, we note that our source localization suggests an origin in the dorsal visual stream and therefore we speculate that the effect may be due to differences in the spatial distribution of information between the two stimulus types. In particular, while global image energy was matched across stimulus classes, the local distribution is likely to have differed substantially between stimulus types. Image energy in the face stimuli is concentrated around the face outline and key feature such as the eyes and hairline, and therefore has predictable spatial structure, but is much more dispersed in the scrambled stimuli and lacking in structure. Therefore, spatial processing of the stimuli in the dorsal stream is likely to be substantially different and might be expected to evoke different neural responses.

### Comparison with previous studies – Gamma

The use of the SAM beamformer to localize the gamma-band response to static images of faces has also been explored in other studies. Gao *et al*. (2012) were able to localize activity to a similar area of right occipito-temporal cortex found in this study. However, they did not find left OFA activity shown here, but instead found some gamma activity in earlier visual areas, particular when only the inner components of the faces were present. A number of studies have looked at the response to static emotional faces (Luo *et al*., 2007, 2009; Maratos *et al*., 2012) and found the strongest activity in relatively early, medial areas of visual cortex.

A comparison of the current study with this prior research points to two important methodological advances used here. First, in previous studies, statistical comparisons have been between activity during face presentation and the baseline period (or in the case of Maratos *et al*. (2012) between activity during presentation of different emotional expressions). Such comparisons do not match the low-level image properties of the stimuli and therefore differences found may reflect responses to low-level image features in addition to (or instead of) face-specific responses. This would explain why these previous studies found activity in early visual areas that are known to be sensitive to low-level image features but are generally not thought to be specific to face processing. Indeed, when we performed a comparison of the response to face trials vs. baseline using our current data, we found gamma activity widespread throughout occipital cortex with the most significant differences occurring around striate cortex and neighbouring areas (Fig.3). Thus, it is highly recommended that in future MEG studies researchers should aim to contrast face stimuli with control images that are as closely matched for low-level stimulus properties as possible.

Secondly, it can be seen that some of these previous studies have tended to use frequency bandwidths that, while consistent with previous studies of the visual gamma response using grating stimuli (Muthukumaraswamy *et al*., 2010), were not, on the basis of the current study, optimal for finding that part of the gamma response sensitive to the presence of faces. In particular, the 30–50 Hz bandwidth employed by Luo *et al*. (2007, 2009) would have filtered out much of the face-specific response found here.

Likewise, a comparison between Figs2 and 6 demonstrates that while it is possible to measure face-specific effects using relatively broadband filtering encompassing the whole gamma frequency range, greater statistical sensitivity can be achieved by restricting analysis to that part of the gamma band for which the response is truly face-specific (approximately 50–90 Hz based on the evidence found here).

### Comparison with previous studies – M170

Unlike the gamma response, the M170 ERF seems to be highly specific to faces and is largely attenuated for non-face stimuli. Thus, it seems likely that the M170 can be well localized without the use of a matched control stimulus. However, we note that the beamfomer method used here is incompatible with the use of permutation testing of a single condition: a single state source analysis can only produce positive values of source power, while the one-sample permutation test requires participant-level measures to be able to take both signs (due to the use of sign-flipping to compute the null distribution of the test statistic). For this reason, it is necessary to contrast the response to faces with a second condition, and a control stimulus matched with faces for low-level image properties may be beneficial in this instance, as ERFs preceding the M170 should theoretically be well matched across conditions (Rossion & Caharel, 2011).

We were able to find three previous studies of the M170 which used beamforming and in which the coordinates of the M170 source were reported (Itier *et al*., 2006; Bayle & Taylor, 2010; Gao *et al*., 2012). Along both the *x-* (posterior-anterior) and the *y-* (left-right) axes our M170 source was within 4 mm of the group mean coordinates from these previous studies. However, along the *z-*axis our source location was approximately 16 mm superior to the group mean. Likewise, our source was 13 mm superior to the centroid of the face-specific N200 (the intracranial analogue of the M170) found by Allison *et al*. (1999), despite our source location matching the centroid to within 1 mm along the other two axes. This suggests that there may have been a superior bias in our source localization.

This bias could have been due to the beamformer incorrectly combining two correlated sources, one in FFA and another in a superior part of temporal cortex (such as STS perhaps), to a single source in an intermediate location. However, it is not clear why such a bias would not also have been present in previous beamforming studies.

Alternatively, it is possible that differences between our source localization and those found in previous studies represent stochastic variation around the true source location. Consistent with this, when averaged across all four studies (the current study and the three prior studies) the mean source location of the M170 given by the beamformer was within 3.5 mm of the centroid found by Allison *et al*. (1999) – whereas source locations from individual studies were all > 11 mm from the centroid – suggesting convergence across experiments towards the intracranial findings.

## Conclusions

We have demonstrated the validity of MEG, combined with the SAM beamfomer, to measure and localize both induced and evoked responses to faces. Our results, particularly with respect to the gamma-band response and M170, are strongly consistent with data from fMRI on the location of cortical areas involved in face processing and data from intracranial recordings in humans on the temporal and time–frequency aspects of this response. We have also elucidated some important methodological points with respect to beamformer imaging of faces: namely that contrasts with appropriate control stimuli and careful selection of frequency bands of interest are necessary to maximize statistical power of the beamfomer. Incidental to this, we have produced the novel finding that face-specific responses are specific to a sub-band (50–90 Hz) of the broader gamma-band response.

Previously, combined data on the spatial, temporal and frequency characteristics of the face-specific response have come from intracranial EEG studies. While we do not dispute that such studies must remain the ‘gold standard’ in this field (not least because they provide necessary ‘ground truth’ data with which to validate MEG source reconstruction) we note that, as an experimental technique, MEG is much more suited to high-throughput experimental testing and/or testing of selectively defined populations (for instance in those with conditions such as prosopagnosia or autism). Furthermore, modern MEG systems have the advantage of whole-head coverage, meaning that they can be used to study extended brain networks in a manner that generally cannot be achieved using intracranial methods. For this reason, we believe that the potential for MEG to measure cortical responses to faces, as demonstrated in this and previous studies, offers exciting new avenues for research into the cortical basis of face perception.

## Abbreviations

ERF: event-related field
ERP: event-related potential
FFA: fusiform face area
fMRI: functional magnetic resonance imaging
GFP: global field power
MEG: magnetoencephalography
OFA: occipital face area
RMS: root mean square
SAM: synthetic aperture magnetometry
STS: superior temporal sulucus
TFWOI: time-frequency window of interest
TWOI: time window of interest
